# Nomograms for Predicting High Hospitalization Costs and Prolonged Stay among Hospitalized Patients with pAECOPD

**DOI:** 10.1155/2024/2639080

**Published:** 2024-09-06

**Authors:** Nafeisa Dilixiati, Mengyu Lian, Ziliang Hou, Jie Song, Jingjing Yang, Ruiyan Lin, Jinxiang Wang

**Affiliations:** Department of Pulmonary and Critical Care Medicine Beijing Luhe Hospital Capital Medical University, Beijing, China

## Abstract

This study aimed to develop nomograms to predict high hospitalization costs and prolonged stays in hospitalized acute exacerbations of chronic obstructive pulmonary disease (AECOPD) patients with community-acquired pneumonia (CAP), also known as pAECOPD. A total of 635 patients with pAECOPD were included in this observational study and divided into training and testing sets. Variables were initially screened using univariate analysis, and then further selected using a backward stepwise regression. Multivariable logistic regression was performed to establish nomograms. The predictive performance of the model was evaluated using the receiver operating characteristic (ROC) curve, area under the curve (AUC), calibration curve, and decision curve analysis (DCA) in both the training and testing sets. Finally, the logistic regression analysis showed that elevated white blood cell count (WBC>10 × 10^9^ cells/l), hypoalbuminemia, pulmonary encephalopathy, respiratory failure, diabetes, and respiratory intensive care unit (RICU) admissions were risk factors for predicting high hospitalization costs in pAECOPD patients. The AUC value was 0.756 (95% CI: 0.699–0.812) in the training set and 0.792 (95% CI: 0.718–0.867) in the testing set. The calibration plot and DCA curve indicated the model had good predictive performance. Furthermore, decreased total protein, pulmonary encephalopathy, reflux esophagitis, and RICU admissions were risk factors for predicting prolonged stays in pAECOPD patients. The AUC value was 0.629 (95% CI: 0.575–0.682) in the training set and 0.620 (95% CI: 0.539–0.701) in the testing set. The calibration plot and DCA curve indicated the model had good predictive performance. We developed and validated two nomograms for predicting high hospitalization costs and prolonged stay, respectively, among hospitalized patients with pAECOPD. This trial is registered with ChiCTR2000039959.

## 1. Introduction

Chronic obstructive pulmonary disease (COPD) is a chronic respiratory disorder characterized by persistent airflow obstruction and chronic respiratory symptoms [1]. It has emerged as the third leading cause of global disease-related mortality and 90% of these deaths occur in low- and middle-income countries [2, 3]. Acute exacerbation of chronic obstructive pulmonary disease (AECOPD) is associated with increased airway and systemic inflammation [4], oxidative stress [5], and destruction of airway and alveolar structures [6]. Moreover, it contributes to accelerated deterioration of lung function [7], increased mortality rates [8], and is the main cause of hospitalization [9] and high medical costs [1] for COPD patients, and imposing significant social and economic burdens.

Common triggers for AECOPD include infections, allergens, and air pollution [1]. Respiratory tract infections account for approximately 50% to 75% of AECOPD cases [10]. Our prior research has revealed that approximately 31% of hospitalized AECOPD patients experience concurrent community-acquired pneumonia (CAP), also known as pAECOPD, and this subgroup of patients tends to have prolonged hospital stays and higher healthcare expenses [11]. Studies have also indicated higher rates of intensive care unit (ICU) admissions and the need for assisted ventilation among pAECOPD patients compared to AECOPD patients without pneumonia (npAECOPD) [12]. CAP has been identified as an independent risk factor for in-hospital mortality in critically ill AECOPD patients [13]. These studies collectively highlight the exacerbation of economic burdens and adverse outcomes associated with CAP in AECOPD patients.

Although several studies have investigated the factors associated with increased hospital costs and prolonged stays in AECOPD patients, few studies have specifically focused on identifying risk factors for higher healthcare expenses and extended hospitalization in pAECOPD patients. Therefore, the aim of our retrospective study is to identify these risk factors and develop predictive models that can facilitate targeted interventions in hospitalized pAECOPD patients. This research endeavor aims to provide evidence-based guidance for clinical decision-making and timely interventions tailored to the needs of pAECOPD patients.

## 2. Materials and Methods

### 2.1. Population and Study Design

A retrospective analysis was conducted on patients with pAECOPD admitted to the Pulmonary and Critical Care Medicine Department at Beijing Luhe Hospital, Capital Medical University, from January 2012 to December 2019. Exclusion criteria include the following: patients who were nondischarged against medical advice and hospitalized for less than 24 h . The study was approved by the Ethics Committee of Beijing Luhe Hospital, Capital Medical University (No: 2020-LHKY-014-03), and conducted in accordance with the principles of the Helsinki Declaration. As this study was a retrospective observational study using anonymous data, informed consent exemption was obtained from the Ethics Committee.

### 2.2. Data Collection and Definitions

The following clinical data were collected: the demographic characteristics and general clinical data include gender, age, smoking status, duration of COPD, home oxygen therapy, home noninvasive ventilation, inhaled pharmacological therapy, previous hospitalization history due to COPD in the past year, and age-adjusted Charlson comorbidity index (aCCI) [14]. Respiratory system-related complications and comorbidities are pleural effusion, respiratory failure, pulmonary encephalopathy, chronic cor pulmonale, asthma, bronchiectasis, and obstructive sleep apnea hypopnea syndrome. Common comorbidities outside the respiratory system are coronary heart disease, hypertension, chronic congestive heart failure, diabetes mellitus, chronic renal diseases, and reflux esophagitis. Routine laboratory examination data included cardiac ultrasound indicators which are as follows: pulmonary artery pressure and left ventricular ejection fraction. Number of days with acute exacerbation, length of hospital stays, respiratory intensive care unit (RICU) admissions, hospitalization costs, medical insurance coverage, and major medications during hospitalization were also included.

Anthonisen type: AECOPD can be classified into three types based on the presence and combination of three symptoms: increased breathlessness, increased sputum volume, and increased purulence. Type I exacerbation is defined as the simultaneous occurrence of all three symptoms. Type II exacerbation is defined as the presence of two out of the three symptoms. Type III exacerbation is defined when only one symptom is present [15].

Diagnosis criteria: the diagnosis of COPD is established according to the Global Initiative for Chronic Obstructive Lung Disease (GOLD) strategy [16]. AECOPD is defined as the occurrence of worsening dyspnea, intensified coughing, increased sputum volume, and purulent sputum that exceed the patient's daily variability, resulting in the need for treatment plan modification or even hospitalization.

The diagnosis criteria of CAP are based on the diagnosis and treatment guidelines for CAP in adults: 2016 clinical practice guidelines by the Chinese Thoracic Society, Chinese Medical Association [17].

The primary outcome of the study is the cost of hospitalization, which refers to the aggregate medical expenditures borne by patients, encompassing both personal out-of-pocket expenses and/or payments made through medical insurance. High hospitalization cost was defined as exceeding the third quartile value of the study population.

The secondary outcome of the study is the duration of hospital stay, which is measured as the interval in days between admission and discharge. Prolonged hospital stay was defined as exceeding the third quartile value of the study population [18].

### 2.3. Statistical Analysis

Statistical analysis was performed using R Studio (version 4.1.2). Multiple imputation methods were utilized to fill in missing data for variables with less than 30% missingness. Laboratory test data in the independent variables were treated as categorical variables based on the reference values provided by the laboratory. Age-adjusted Charlson comorbidity index was divided into two groups: <6 and ≥6, following the previous literature [19]. All included patients were randomly divided into a training set and a validation set in a 7 : 3 ratio. Categorical variables were presented as numbers (percentages) and analyzed using the chi-squared test. Continuous variables were described as mean (standard deviation) if they followed a normal distribution and analyzed using the *t*-test. Skewed distributed continuous variables were described as median (interquartile range) and analyzed using the Mann–Whitney *U* test.

Variables were initially screened using the univariate analysis, and then further selected using a backward stepwise regression method based on the minimum Akaike information criterion (AIC) value. Multivariable logistic regression was performed to establish a predictive model, and a nomogram was plotted. The predictive performance of the model was evaluated using receiver operating characteristic (ROC) curves, area under the curve (AUC), calibration curves, and decision curve analysis (DCA) in both the training and testing sets. A significance level of *p* < 0.05 was considered statistically significant.

## 3. Results

### 3.1. Baseline Characteristics of the Subjects

**Figure 1:**
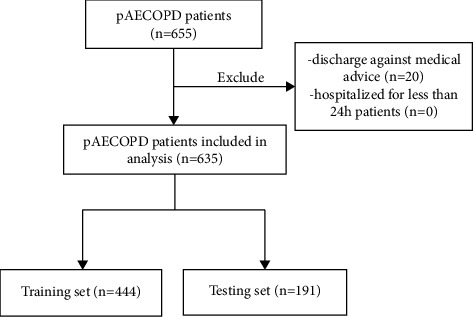
Study flow diagram. pAECOPD: acute exacerbations of chronic obstructive pulmonary disease with community-acquired pneumonia.

A total of 655 pAECOPD patients admitted to the Pulmonary and Critical Care Medicine Department of Beijing Luhe Hospital, Capital Medical University, from June 2012 to June 2019, were collected. After excluding 20 patients based on exclusion criteria, a final cohort of 635 pAECOPD patients was included in the study (Figure 1). The median age was 75.0 (69.00, 81.00) years, with 446 males and 189 females. The 635 patients were randomly divided into a training set and a testing set in a ratio of 7 : 3. The training set consisted of 444 patients, accounting for 70.0% of the total, with 317 males and 127 females. The validation set included 191 patients, accounting for 30.0% of the total, with 129 males and 62 females. The supplementary material provides the clinical characteristics of the subjects in the training set and testing set. A comparative analysis between the two groups indicated good comparability among the patients.

### 3.2. Development and Validation of a Nomogram for Predicting High Hospitalization Costs in pAECOPD Patients

**Figure 2:**
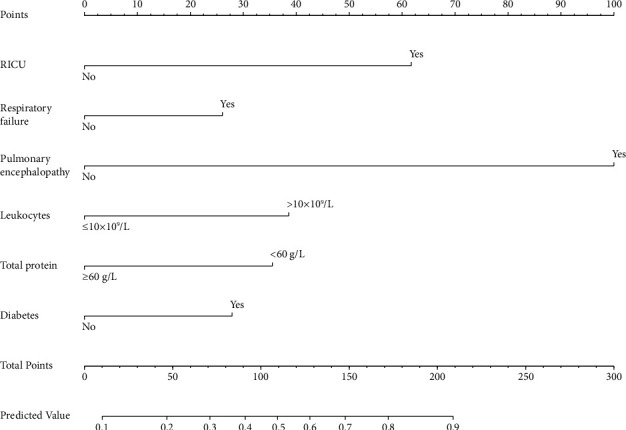
A nomogram for predicting high hospitalization costs of pAECOPD patients. RICU: respiratory intensive care unit.

In the training set, a preliminary variable selection was performed using the univariate analysis, resulting in the identification of 17 variables (Table 1). Subsequently, a backward stepwise regression method based on the AIC criterion was applied to further refine the variable selection, resulting in the inclusion of 9 variables (AIC = 416.42). These 9 variables were then incorporated into a multivariable logistic regression analysis. Eventually, 6 variables were determined as predictive factors for high hospitalization costs in pAECOPD patients. These variables included elevated white blood cell count (WBC > 10 × 10^9^ cells/l), hypoalbuminemia, pulmonary encephalopathy, respiratory failure, diabetes, and RICU admissions (Table 1). A prediction model was fitted using these predictive factors, and a nomogram was generated (Figure 2).

**Figure 3:**
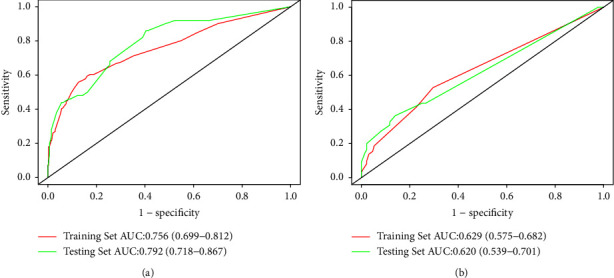
The ROC curves of a nomogram for predicting high hospitalization costs of pAECOPD patients (a). The ROC curves of a nomogram for predicting prolonged hospital stay of pAECOPD patients (b).

**Figure 4:**
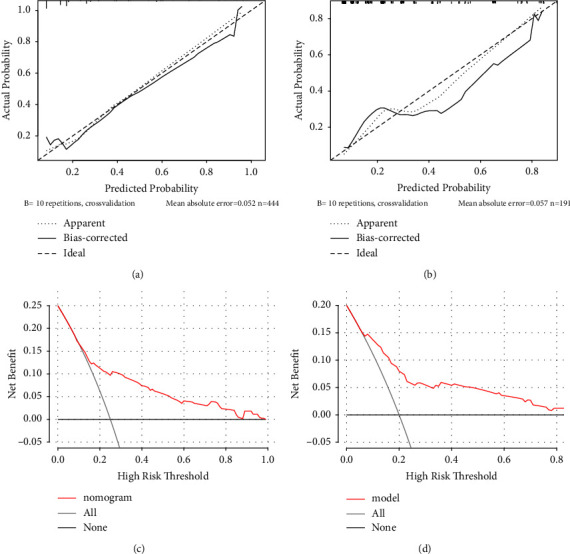
Calibration curves of a nomogram for predicting high hospitalization costs of pAECOPD patients: (a) training set and (b) testing set. Decision curve analysis of a nomogram for predicting high hospitalization costs of pAECOPD patients: (c) training set and (d): testing set.

In the training set, the prediction model exhibited an AUC value of 0.756 (95% CI: 0.699–0.812) and a Brier score of 0.144, indicating good accuracy of the prediction model (Figure 3(a)). The model had a sensitivity of 0.595, specificity of 0.838, and Youden's index of 0.268. The calibration curve showed no significant deviation from the reference line, suggesting good calibration of the model in this cohort (Figure 4(a)). The decision curve analysis (DCA) curve demonstrated a favorable overall net benefit over a wide range of threshold probabilities (Figure 4(c)).

In the testing set, the prediction model achieved an AUC value of 0.792 (95% CI: 0.718–0.867) with a Brier score of 0.144, indicating slightly improved accuracy compared to the training set (Figure 3(a)). The model had a sensitivity of 0.860, specificity of 0.596, and Youden's index of 0.175. The calibration curve and DCA curve in the validation dataset were similar to those in the training set (Figures 4(b) and 4(d)).

### 3.3. Development and Validation of a Nomogram for Predicting Prolonged Length of Stay in pAECOPD Patients

**Figure 5:**
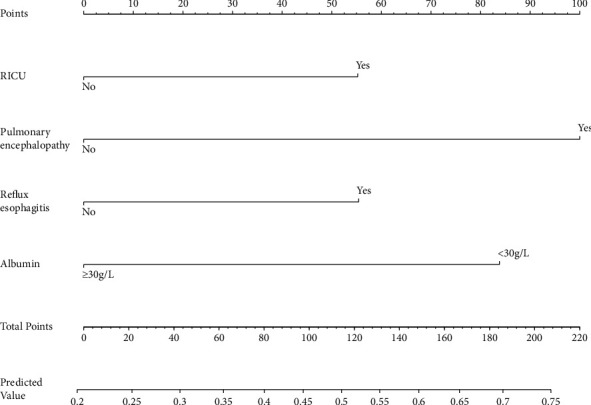
A nomogram for predicting prolonged hospital stay of pAECOPD patients. RICU: respiratory intensive care unit.

In the training set, variables were initially screened through the univariate analysis, resulting in the selection of 8 variables (Table 2). Subsequently, a stepwise backward regression method based on the AIC criterion was employed to further select variables, resulting in the selection of 5 variables (AIC = 505.54). These 5 variables were included in the multiple logistic regression model, and ultimately, 4 variables were identified as predictive factors, namely, decreased total protein, pulmonary encephalopathy, reflux esophagitis, and RICU admissions (Table 2). The model was fitted using these predictive factors, and a nomogram was generated (Figure 5).

**Figure 6:**
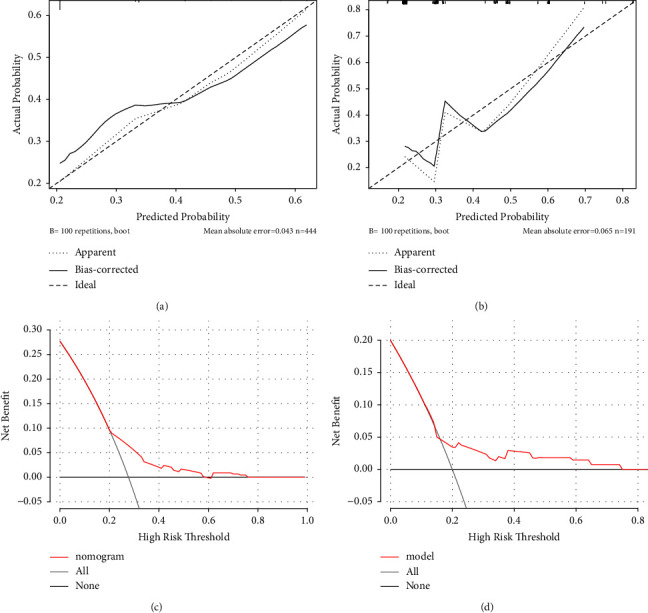
Calibration curves of a nomogram for predicting prolonged hospital stay of pAECOPD patients: (a) training set and (b) testing set. Decision curve analysis of a nomogram for predicting prolonged hospital stay of pAECOPD patients: (c) training set and (d) testing set.

In the training set, the predictive model demonstrated an AUC value of 0.629 (95% CI: 0.575–0.682) and a Brier score of 0.187 (Figure 3(b)). The model had a sensitivity of 0.528, specificity of 0.704, and Youden's index of 0.269. The calibration curve showed no significant deviation from the reference line, indicating good consistency of the cohort (Figure 6(a)). The DCA curve demonstrated that the model had favorable overall net benefit within a certain threshold probability range (Figure 6(c)).

In the testing set, the predictive model exhibited an AUC value of 0.620 (95% CI: 0.539–0.701) and a Brier score of 0.187, indicating similar accuracy to the training set (Figure 3(b)). The model had a sensitivity of 0.364, specificity of 0.860, and Youden's index of 0.313. The calibration curve and DCA curve were like those of the training set (Figures 6(b) and 6(d)).

## 4. Discussion

Our research has found that elevated white blood cell count, hypoalbuminemia, pulmonary encephalopathy, respiratory failure, diabetes, and RICU admissions are risk factors for high hospitalization costs in patients with pAECOPD. In addition, we have also identified decreased total protein, pulmonary encephalopathy, reflux esophagitis, and RICU admissions as risk factors for prolonged hospital stay in pAECOPD patients. Based on these risk factors, we have developed predictive models for both high hospitalization costs and prolonged length of stay in pAECOPD patients and have plotted nomograms. The models have been validated and have shown good discriminatory performance.

In our study, the median length of hospital stay for pAECOPD patients was 10 days (IQR: 8–13), which is similar with previous studies conducted in China [13, 20–22]. However, it is higher than findings from three studies conducted in Norway [23], Israel [24], and Denmark [12], respectively. We hypothesize that these differences may be attributed to variances in healthcare system management among different countries.

Studies [18, 25] have reported a correlation between hypoalbuminemia and prolonged hospitalization in COPD and CAP patients, which aligns with our research findings. This association may be attributed to the involvement of serum albumin in immune and defense processes, and a decrease in serum albumin weakens these processes, leading to compromised immune and defense capabilities [26]. In addition, hypoalbuminemia in critically ill patients can impact the protein-binding rate of certain antimicrobial drugs, increasing drug clearance [27] and potentially resulting in inadequate treatment [28] and prolonged hospital stays.

The level of total serum protein can reflect the nutritional status of patients. For critically ill patients, energy expenditure will increase under stress, and malnutrition and/or hypoproteinemia are prone to occur. A study had shown that undernourished COPD patients cost more than well-nourished patients [29], which is similar to our result. Moreover, chronic malnutrition was associated with poor lung function in COPD patients [30]. Efthimiou et al. showed that after nutritional support, malnourished COPD patients had improved respiratory muscle strength and handgrip strength, improved dyspnea, and increased 6-minute walking distance [31]. Elevated leukocyte counts, known as inflammatory cells that play a crucial role in mediating the inflammatory response, were found to be associated with higher hospital costs. Clinical guidelines commonly recommend administering antimicrobial drugs in AECOPD patients based on increased leukocyte counts [32].

Our research findings indicate a strong correlation between the combination of diabetes and pAECOPD and high hospitalization costs, which is consistent with a study conducted in China on the hospitalization costs of AECOPD patients [33]. Diabetic patients experience various mechanisms, such as impaired neutrophil function [34], compromised antioxidant system, and humoral immunity [35], leading to immune dysfunction and increased susceptibility to infections in the lungs as well as other body parts [36]. Consequently, they require more treatments and incur higher expenses. Both stress and inflammation can cause fluctuations in blood glucose levels among AECOPD patients [37]. Moreover, the administration of corticosteroids during hospitalization for pAECOPD may contribute to elevated blood glucose levels [38]. In our previous research, 30% of pAECOPD patients received intravenous corticosteroid treatment [11]. Therefore, it is essential to emphasize blood glucose control for pAECOPD patients.

Reflux esophagitis is frequently observed in COPD patients, with an estimated prevalence ranging from 17% to 54% [39]. The frequency of gastric reflux symptoms was significantly correlated with the decrease of FEV_1_ [40]. Our research findings indicate that concomitant reflux esophagitis is a significant risk factor for prolonged hospitalization in pAECOPD patients. The pathophysiological relationship between AECOPD and reflux esophagitis is currently under investigation. Proposed mechanisms include microaspiration of gastric contents leading to airway irritation and increased airway resistance, vagally mediated bronchoconstriction, and heightened bronchial responsiveness to other stimuli due to esophageal acid exposure [41]. Moreover, reflux esophagitis predisposes patients to recurrent aspirations, leading to aspiration pneumonia [42]. Patients with aspiration pneumonia experience longer hospital stays and higher mortality rates compared to those with nonaspiration pneumonia [43]. We speculate that the prolonged hospitalization duration in pAECOPD patients with concomitant reflux esophagitis may be attributed to a higher incidence of aspiration pneumonia within the CAP subtype. However, since our study was retrospective, we could not definitively determine whether the CAP cases included in the study were specifically related to aspiration pneumonia. Future prospective research is warranted to elucidate this further.

In study, we identified respiratory failure and hypercapnic encephalopathy as significant risk factors for increased hospitalization costs in patients with pAECOPD. These factors are likely attributed to the need for respiratory support and a higher rate of ICU admissions [1]. Hypercapnic encephalopathy was also associated with prolonged hospitalization duration, confirming the findings of Dong et al. [44]. This association can be explained by the presence of severe respiratory muscle fatigue and inadequate ventilation in patients with hypercapnic encephalopathy, which require prolonged respiratory support. Consistent with our study, admission to the RICU was associated with longer hospital stays and higher healthcare expenses.

However, our study had several limitations. Firstly, it was conducted at a single center without external validation, although internal validation was performed using a testing set. Secondly, due to a high proportion of missing lung function data, it was not included in the analysis. However, we included arterial blood gas analysis parameters, respiratory failure, and hypercapnic encephalopathy as variables to capture disease severity, partially compensating for the impact of missing lung function data on the model. Thirdly, in this study, the AUC value of the prediction model for prolonged hospital stay in pAECOPD patients was close to 0.6 and the predictive value is limited. However, we found four independent risk factors for prolonged hospital stay in pAECOPD patients, all with *p* values less than 0.05. By targeting the aforementioned four risk factors, clinicians can enhance management practices and reduce hospital stay for AECOPD patients. Lastly, our study only considered direct hospitalization costs in pAECOPD patients and did not assess indirect healthcare expenses. Future research should be aimed to further validate our findings, address the aforementioned limitations, and include more clinical characteristics, and refine our nomograms.

## 5. Conclusion

We have developed nomograms for predicting high hospitalization costs and prolonged hospital stay in pAECOPD patients, considering six risk factors (elevated white blood cell count, hypoalbuminemia, pulmonary encephalopathy, respiratory failure, diabetes, and RICU admissions) for high hospitalization costs and four risk factors (decreased total protein, pulmonary encephalopathy, reflux esophagitis, and RICU admissions) for prolonged hospital stay. Utilizing the risk factors and predictive nomograms can help clinicians identify patients at risk for prolonged hospital stays and increased hospitalization costs. This enables timely intervention and management, and conserving health resources.

## Figures and Tables

**Table 1:** Prognostic factors associated with high hospital costs in pAECOPD patients.

Variables	Univariate		Multivariate	
	OR (95% CI)	*p* value	OR (95% CI)	*p* value
Duration of COPD	1.018 (1.002, 1.035)	0.03		
Pulmonary encephalopathy	15.919 (5.775, 56.133)	<0.001	9.12 (2.90, 35.3)	<0.001
Respiratory failure	3.548 (2.279, 5.576)	<0.001	1.78 (1.04, 3.02)	0.033
Hydrothorax	1.961 (1.182, 3.221)	0.008		
Cor pulmonale	1.617 (1.045, 2.529)	0.033		
Chronic cardiac failure	1.691 (1.097, 2.610)	0.017		
Diabetes	2.047 (1.231, 3.371)	0.005	1.85 (1.03, 3.30)	0.038
aCCI ≥6 score	1.625 (1.055, 2.508)	0.028		
Leukocytes >10 × 10^9^/L	2.301 (1.366, 3.840)	0.002	2.35 (1.30, 4.20)	0.004
Eosinophils <0.02 × 10^9^/L	1.956 (1.156, 3.267)	0.011		
TP ≥60 g/L	2.129 (1.380, 3.304)	0.001	2.19 (1.32, 3.66)	0.002
Alb <30 g/L	2.636 (1.298, 5.280)	0.006		
Ca ≥2.11 mmol/L	0.587 (0.380, 0.904)	0.016		
P <0.85 mmol/L	1.961 (1.122, 3.374)	0.016		
LDH >250 U/L	2.446 (1.318, 4.481)	0.004		
PaCO_2_ >50 mmHg	1.846 (1.188, 2.864)	0.006		
RICU	6.941 (3.984, 12.315)	<0.001	3.91 (2.06, 7.50)	<0.001

COPD, chronic obstructive pulmonary disease; AECOPD, acute exacerbations of COPD; pAECOPD, pneumonia-complicating AECOPD; OR, odds ratio; 95% CI, 95% confidence interval; aCCI, age-adjusted Charlson comorbidity index; TP, total protein; Alb, albumin; Ca: calcium; P: phosphorus; LDH, lactate dehydrogenase; PaCO_2_, arterial carbon dioxide partial pressure; RICU, respiratory intensive care unit.

**Table 2:** Prognostic factors associated with prolonged LOS in pAECOPD patients.

Variables	Univariate		Multivariate	
	OR (95% CI)	*p* value	OR (95% CI)	*p* value
Pulmonary encephalopathy	4.10 (1.72, 10.18)	0.002	3.26 (1.30, 8.50)	0.013
Respiratory failure	1.74 (1.14, 2.65)	0.011		
Hydrothorax	1.74 (1.05, 2.83)	0.028		
RE	1.76 (1.03, 2.96)	0.034	1.93 (1.11, 3.30)	0.018
CRP	1.75 (1.11, 2.82)	0.019		
TP	1.53 (1.01, 2.33)	0.045		
Alb	2.55 (1.27, 5.10)	0.008	2.69 (1.31, 5.49)	0.006
RICU	2.44 (1.41, 4.20)	0.001	1.92 (1.06, 3.43)	0.028

LOS, length of stay; COPD, chronic obstructive pulmonary disease; AECOPD, acute exacerbations of COPD; pAECOPD, pneumonia-complicating AECOPD; OR, odds ratio; 95% CI, 95% confidence interval; RE, reflux esophagitis; CRP, C-reactive protein; TP, total protein; Alb, albumin; RICU, respiratory intensive care unit.

## Data Availability

The datasets used in the current study are available from the corresponding author on reasonable request.

## Supplementary Materials

Supplementary Materials Supplementary material Table S1: demographic and baseline clinical characteristics of pAECOPD patients.
