# Supplementary material for: Nomograms for Predicting High Hospitalization Costs and Prolonged Stay among Hospitalized Patients with pAECOPD
Source: Can Respir J. 2024 Sep 6;2024:2639080. doi: 10.1155/2024/2639080 (PMC11398965; doi:10.1155/2024/2639080)
Supplement: Supplementary Materials — Supplementary material Table S1: demographic and baseline clinical characteristics of pAECOPD patients. [file 2639080.f1.docx]

**Supplementary material 1 Demographic and baseline clinical characteristics of pAECOPD patients**

| **Characteristics** | **Overall**  (n = 635) | | | **Training Set**  (n = 444) | **Testing Set**  (n = 191) | | ***P* value** | | |
| --- | --- | --- | --- | --- | --- | --- | --- | --- | --- |
| Sex |  | |  | |  | |  | | |
| Male | 446 (70.2) | | 317 (71.4) | | 129 (67.5) | | | 0.379 | |
| Female | 189 (29.8) | | 127 (28.6) | | 62 (32.5) | |  | | |
| Age, years | 75.00 [69.00, 81.00] | | 75.00 [69.00, 81.00] | | 75.00 [69.00, 81.00] | | | 0.628 | |
| Smoking history |  | |  | |  | | |  | |
| Current smoker | 134 (21.1) | | 95 (21.4) | | 39 (20.4) | | | 0.889 | |
| Ex-smoker | 362 (57.0) | | 254 (57.2) | | 108 (56.5) | | |  | |
| Non-smoker | 139 (21.9) | | 95 (21.4) | | 44 (23.0) | | |  | |
| Duration of COPD, years | 13.00 [9.00, 30.00] | | 12.00 [9.00, 30.00] | | 14.00 [9.00, 30.00] | | | 0.962 | |
| inhaled pharmacological therapy | 355 (55.9) | | 247 (55.6) | | 108 (56.5) | | | 0.9 | |
| LTOT | 58 (9.1) | | 42 (9.5) | | 16 (8.4) | | | 0.776 | |
| Home NIV | 39 (6.1) | | 23 (5.2) | | 16 (8.4) | | | 0.174 | |
| Medical insurance | 585 (92.1) | | 409 (92.1) | | 176 (92.1) | | | 1 | |
| Days of exacerbation, day | 5.00 [3.00, 7.00] | | 5.00 [3.00, 7.00] | | 5.00 [2.50, 7.50] | | | 0.773 | |
| First admission for AECOPD | 110 (17.3) | | 66 (14.9) | | 44 (23.0) | | | 0.017 | |
| Anthonisen type |  | |  | |  | | |  | |
| type I | 249 (39.2) | | 176 (39.6) | | 73 (38.2) | | | 0.903 | |
| type II | 274 (43.1) | | 189 (42.6) | | 85 (44.5) | | |  | |
| type III | 112 (17.6) | | 79 (17.8) | | 33 (17.3) | | |  | |
| Hydrothorax | 136 (21.4) | | 89 (20.0) | | 47 (24.6) | | | 0.238 | |
| Respiratory failure | 242 (38.1) | | 167 (37.6) | | 75 (39.3) | | | 0.761 | |
| Pulmonary encephalopathy | 36 (5.7) | | 22 (5.0) | | 14 (7.3) | | | 0.317 | |
| Chronic cor pulmonale | 336 (52.9) | | 241 (54.3) | | 95 (49.7) | | | 0.335 | |
| Asthma | 78 (12.3) | | 56 (12.6) | | 22 (11.5) | | | 0.8 | |
| Bronchiectasis | 151 (23.8) | | 110 (24.8) | | 41 (21.5) | | | 0.426 | |
| OSAHS | 18 (2.8) | | 15 (3.4) | | 3 (1.6) | | | 0.318 | |
| CHD | 211 (33.2) | | 154 (34.7) | | 57 (29.8) | | | 0.273 | |
| Hypertension | 194 (30.6) | | 137 (30.9) | | 57 (29.8) | | | 0.873 | |
| Congestive heart failure | 253 (39.8) | | 185 (41.7) | | 68 (35.6) | | | 0.179 | |
| Diabetes | 128 (20.2) | | 87 (19.6) | | 41 (21.5) | | | 0.666 | |
| Chronic renal diseases | 45 (7.1) | | 33 (7.4) | | 12 (6.3) | | | 0.727 | |
| RE | 106 (16.7) | | 74 (16.7) | | 32 (16.8) | | | 1 | |
| PTE | 9 (1.4) | | 7 (1.6) | | 2 (1.0) | | | 0.88 | |
| aCCI |  | |  | |  | | |  | |
| <6 score | 364 (57.3) | | 252 (56.8) | | 112 (58.6) | | | 0.725 | |
| ≥6 score | 271 (42.7) | | 192 (43.2) | | 79 (41.4) | | |  | |
| Fever | | 144 (22.7) | | 96 (21.6) | | 48 (25.1) | | | 0.387 |
| EF, % | | |  |  | |  |  | | |
| <50 | 612 (96.4) | | 425 (95.7) | | 187 (97.9) | | | 0.263 | |
| ≥50 | 23 (3.6) | | 19 (4.3) | | 4 (2.1) | | |  | |
| Pulmonary artery pressure, mmHg |  | |  | |  | | |  | |
| <25 | 417 (65.7) | | 285 (64.2) | | 132 (69.1) | | | 0.268 | |
| ≥25 | 218 (34.3) | | 159 (35.8) | | 59 (30.9) | | |  | |
| Leukocytes, 10^9^cells/l |  | |  | |  | | |  | |
| ≤10 | 530 (83.5) | | 365 (82.2) | | 165 (86.4) | | | 0.236 | |
| >10 | 105 (16.5) | | 79 (17.8) | | 26 (13.6) | | |  | |
| Eosinophils, % |  | |  | |  | | |  | |
| <0.5 | 111 (17.5) | | 80 (18.0) | | 31 (16.2) | | | 0.574 | |
| 0.5-5 | 509 (80.2) | | 352 (79.3) | | 157 (82.2) | | |  | |
| >5 | 15 (2.4) | | 12 (2.7) | | 3 (1.6) | | |  | |
| Platelets, 10^9^ cells/L |  | |  | |  | | |  | |
| <100 | 608 (95.7) | | 425 (95.7) | | 183 (95.8) | | | 1 | |
| ≥100 | 27 (4.3) | | 19 (4.3) | | 8 (4.2) | | |  | |
| CRP, mg/L |  | |  | |  | | |  | |
| ≤10 | 224 (35.3) | | 150 (33.8) | | 74 (38.7) | | | 0.267 | |
| >10 | 411 (64.7) | | 294 (66.2) | | 117 (61.3) | | |  | |
| TP, g/L |  | |  | |  | | |  | |
| <60 | 359 (56.5) | | 254 (57.2) | | 105 (55.0) | | | 0.665 | |
| ≥60 | 276 (43.5) | | 190 (42.8) | | 86 (45.0) | | |  | |
| Alb, g/L |  | |  | |  | | |  | |
| <30 | 581 (91.5) | | 408 (91.9) | | 173 (90.6) | | | 0.696 | |
| ≥30 | 54 (8.5) | | 36 (8.1) | | 18 (9.4) | | |  | |
| ALT, U/L |  | |  | |  | | |  | |
| ≤50 | 613 (96.5) | | 431 (97.1) | | 182 (95.3) | | | 0.373 | |
| >50 | 22 (3.5) | | 13 (2.9) | | 9 (4.7) | | |  | |
| Cr, µmol/L |  | |  | |  | | |  | |
| ≤97 | 562 (88.5) | | 392 (88.3) | | 170 (89.0) | | | 0.901 | |
| >97 | 73 (11.5) | | 52 (11.7) | | 21 (11.0) | | |  | |
| Urea, mmol/L |  | |  | |  | | |  | |
| <8 | 538 (84.7) | | 378 (85.1) | | 160 (83.8) | | | 0.75 | |
| ≥8 | 97 (15.3) | | 66 (14.9) | | 31 (16.2) | | |  | |
| Ca, mmol/L |  | |  | |  | | |  | |
| <2.11 | 288 (45.4) | | 200 (45.0) | | 88 (46.1) | | | 0.879 | |
| ≥2.11 | 347 (54.6) | | 244 (55.0) | | 103 (53.9) | | |  | |
| P, mmol/L |  | |  | |  | | |  | |
| <0.85 | 108 (17.0) | | 68 (15.3) | | 40 (20.9) | | | 0.218 | |
| 0.85-1.51 | 511 (80.5) | | 365 (82.2) | | 146 (76.4) | | |  | |
| >1.51 | 16 (2.5) | | 11 (2.5) | | 5 (2.6) | | |  | |
| LDH, U/L |  | |  | |  | | |  | |
| ≤250 | 561 (88.3) | | 394 (88.7) | | 167 (87.4) | | | 0.738 | |
| >250 | 74 (11.7) | | 50 (11.3) | | 24 (12.6) | | |  | |
| pH |  | |  | |  | | |  | |
| <7.35 | 70 (11.0) | | 49 (11.0) | | 21 (11.0) | | | 0.74 | |
| 7.35-7.45 | 369 (58.1) | | 262 (59.0) | | 107 (56.0) | | |  | |
| >7.45 | 196 (30.9) | | 133 (30.0) | | 63 (33.0) | | |  | |
| PaCO_2_, mmHg, n |  | |  | |  | | |  | |
| ≤50 | 419 (66.0) | | 288 (64.9) | | 131 (68.6) | | | 0.414 | |
| >50 | 216 (34.0) | | 156 (35.1) | | 60 (31.4) | | |  | |
| PaO_2_, mmHg, n |  | |  | |  | | |  | |
| ≥60 | 576 (90.7) | | 402 (90.5) | | 174 (91.1) | | | 0.941 | |
| <60 | 59 (9.3) | | 42 (9.5) | | 17 (8.9) | | |  | |
| LOS | 10.00 [8.00, 13.00] | | 10.00 [7.75, 13.00] | | 10.00 [8.00, 13.00] | | | 0.763 | |
| RICU admission, n | 95 (15.0) | | 65 (14.6) | | 65 (14.6) | | | 0.822 | |
| Cost of hospitalization, RMB | 15813.48 [12427.94, 21361.68] | | 16009.16 [12523.46, 21227.25] | | 16009.16 [12523.46, 21227.25] | | | 0.691 | |

**Note**: Continuous variables were summarized as mean (± standard) deviation or median [IQR], and categorical variables as n (%).

**Abbreviations**: COPD, chronic obstructive pulmonary disease; AECOPD, acute exacerbations of COPD; pAECOPD, pneumonia-complicating AECOPD; LTOT, long-term oxygen therapy; NIV, noninvasive ventilation; OSAHS, Obstructive sleep apnea hypopnea syndrome; CHD, coronary heart disease; RE, Reflux Esophagitis; PTE, pulmonary thromboembolism; aCCI, age-adjusted Charlson comorbidity index; EF, ejection fractions; CRP, C-reactive protein; TP, total protein; Alb, albumin; ALT, alanine aminotransferase; Cr, creatinine; Ca: calcium; P: phosphorus; LDH, lactate dehydrogenase; pH, hydrogen ion concentration; PaCO_2_, arterial carbon dioxide partial pressure; PaO_2_, arterial oxygen partial pressure; LOS, length of stay; RICU, respiratory intensive care unit.
